# Changes in the Skin Microbiome Following Dermatological Procedures: A Scoping Review

**DOI:** 10.3390/applmicrobiol4020066

**Published:** 2024-06-18

**Authors:** Jeremy R. Ellis, Eron J. Powell, Luke M. Tomasovic, Rachel L. Marcheskie, Vishruth Girish, Anmol Warman, Darshan Sivaloganathan

**Affiliations:** 1 School of Medicine, Johns Hopkins University, Baltimore, MD 21205, USA;; 2 School of Biological Sciences, The University of Utah, Salt Lake City, UT 84112, USA;; 3 Johns Hopkins Bloomberg School of Public Health, Baltimore, MD 21205, USA;

**Keywords:** skin microbiome, dysbiosis, cutaneous microbiome, dermatological intervention

## Abstract

The skin microbiome consists of bacteria, fungi, viruses, and mites, which play a crucial role in maintaining skin health and immune function. Imbalances in this microbial community, known as dysbiosis, are implicated in various dermatological conditions. While skincare products are known to influence the skin microbiome, the effects of dermatological procedures have not been extensively studied. Here, we perform a scoping review to outline the studies investigating the impacts of dermatological interventions on the skin microbiome. Phototherapy emerged as the most studied intervention, encompassing UV phototherapy, light therapy, laser therapy, and photodynamic therapy. Chemical interventions, such as chemical peels, micropigmentation, and debridement, have comparatively limited studies describing their impacts on the skin microbiome. To date, no studies have been done on a wide variety of common dermatological procedures such as cryotherapy, skin grafts, and dermabrasion, which may have stronger likelihoods of affecting the skin microbiome. This underscores the need for further research on the influences of dermatological procedures, especially chemical and physical interventions, on the skin microbiome. More comprehensive pre-clinical and clinical studies are essential not only for understanding the long-term consequences of these procedures, but also for optimizing patient outcomes in dermatological care.

## 1. Introduction

The skin microbiome comprises the microorganisms that inhabit the human skin. This community includes bacteria, fungi, viruses, and mites, all of which play roles in maintaining skin health [1]. The composition of the microbiome varies with body site, environmental conditions, age, gender, and individual health status [2–4]. Bacteria are the most abundant microbes present on the skin, with key genera including *Staphylococcus*, *Corynebacterium*, and *Propionibacterium* [5]. These bacteria inhabit specific niches on the skin, such as sebaceous glands, hair follicles, and sweat glands [6]. Fungi, primarily of the genus *Malassezia*, are particularly prevalent in sebaceous (oil-producing) areas [7]. Viruses, including bacteriophages that infect bacteria, also form part of the skin microbiome, although they are less studied compared to bacteria and fungi [8]. Additionally, arthropods such as the Demodex mite reside along hair follicles and within sebaceous glands [9].

The skin microbiome is essential for skin health, performing various critical functions. One of its primary roles is protection against pathogens. The microbiome acts as both a physical and chemical barrier, with commensal bacteria competing with pathogenic microbes for resources and space [10]. Bacteria such as *Cutibacterium acnes* produce antimicrobial substances that inhibit pathogen growth [11]. Further, the skin microbiome serves to educate immune responses in the host through microbial metabolites such as free fatty acids, antimicrobial peptides, phenol-soluble modulins, and cell wall components [12]. Dysbiosis, an imbalance or disruption in the native composition of the microbiome, has been implicated in conditions such as atopic dermatitis, which features skin microenvironments with diminished diversity and upregulation of host sensitization to microbe-related antigens [13], whereas conditions such as acne have been linked to overgrowth of *C. acnes* [14].

The skin microbiome also serves to maintain the integrity of the physical skin barrier. Microbes regulate the production of skin lipids that fortify the barrier—for instance, ceramide production is implicated in the retention of moisture and helps prevent deterioration [15]. Certain microbes also play a role in the tight junction formation and function of keratinocytes [16]. Beneficial biofilms also provide protection against pathogens both physically and competitively [17].

From an oversized vantage point, the relationship between humans and the microbes that drift on their skin is symbiotic. These age-old passengers have co-evolved with humans, performing beneficial roles such as breaking down sebum [18,19], maintaining skin pH balance [20–22], and producing antioxidants [23].

Several factors influence the skin microbiome’s composition. Genetic differences such as autoimmune disease predispositions are implicated in individual skin microbiomes [8]. Climate, pollution, hygiene practices, and even clothing impact the microbiome [3,24,25]. Age and hormonal changes, particularly during puberty and senescence, alter the microbial landscape [2,26–28]. Both systemic health conditions such as diabetes and irritable bowel disease, as well as cutaneous conditions like psoriasis and atopic dermatitis can disrupt the skin microbiome [29–31].

Dermatologists have long been aware that certain skincare products also affect microbial balance. Ingredients in soaps, antibiotics, and cosmetics can lead to dysbiosis [4]. However, manipulation of the integrity of the skin’s microbiome due to procedural dermatological interventions, such as excisions, dermabrasion, laser therapy, and other physical procedures, is less understood. Given the importance of the skin microbiome, we seek to define the current scope of the literature exploring skin microbiome alterations caused by dermatologic procedures.

## 2. Methods

A review of the current scope of the literature concerning the effects of dermatological interventions on the skin microbiome was performed by following the guidelines outlined by Arksey and O’Malley [32] and Levac et al. [33]. A scoping review protocol was developed to guide the process and has been registered on protocols.io [34]. It can be accessed at https://dx.doi.org/10.17504/protocols.io.261ge53owg47/v2 (accessed on 16 May 2024). This paper conforms to the PRISMA extension designed for scoping reviews [35].

### 2.1. Eligibility Criteria

Studies were included based on three initial inclusion criteria. The studies must have been published in 2007 or later, as this was the year of the start of the Human Microbiome Project [36]. Studies were selected if they contained the phrase skin microbiome/microbiota/microflora in the title, keywords, or abstract. Note that some studies were included without specifying skin if it was obvious from the context that they were related to it. Studies also were included only if they dealt directly with dermatological interventions. A list of approved dermatological interventions was created by the team using the American Medical Association (AMA)’s current procedural terminology (CPT^®^) codes for dermatology [37]. In total, 23 categories of dermatological procedures were determined. Interventions consisting solely of the use of topical ointments, creams, or emollients were discarded.

Studies were excluded if they were not available in English or if they did not match the overall objectives of the review. Studies dealing with bacteriotherapy and pre/post-biotics were excluded because such treatments are primarily designed to change microbiomes and promote microfloral growth. As mentioned above, permitted studies could not deal with topical creams, ointments, etc. Likewise, studies were excluded if they used oral, intravenous, or any other systemic form of treatment. Finally, studies needed to directly evaluate the effects on the skin microbiome and not involve a treatment for dysbiosis or other related issues. The best studies were those that dealt directly with the effect of a localized dermatological intervention on the skin microbiome.

### 2.2. Study Identification and Selection

A search was performed on PubMed using the advanced search tool on May 12th, 2024. The years included were 2007 to 2030. The search strategy was performed following a similar protocol to a scoping review done by Dol et al. in 2019 [38]. Key terms were selected and included in the search using the advanced search tool as follows: “((acne surgery) OR (excisional biopsy) OR (excision) OR (debride) OR (injection) OR (tattoos) OR (micropigmentation) OR (graft) OR (laser surgery) OR (electrosurgery) OR (cryosurgery) OR (chemosurgery) OR (surgical curettement) OR (phototherapy) OR (dermabrasion) OR (chemical peel) OR (chemical cauterization) OR (mohs) OR (skin peel) OR (electrolysis) OR (radiation)) AND (skin microbiome)”.

After the initial search results, each paper’s title, abstract, and keywords were screened to find papers matching the inclusion criteria. Included papers were then subjected to a full paper review and excluded if they matched the exclusion criteria, following the procedures outlined in the protocol. Papers removed during the full paper review had their exclusion reason noted.

### 2.3. Data Management

Items of interest were put into categories where applicable, approved by the project team, and formatted in a Google document. Included studies were then listed and assessed by two reviewers for each item of interest. If the paper included the item of interest, the correct category was listed underneath it. These data were then moved to a Google sheet for ease of data synthesis. The items of interest were dermatological intervention description; study subject n-value; long- vs short-term (where long-term was defined as a study length lasting more than one week); skin condition; and subject species.

## 3. Results

**Figure 1.**
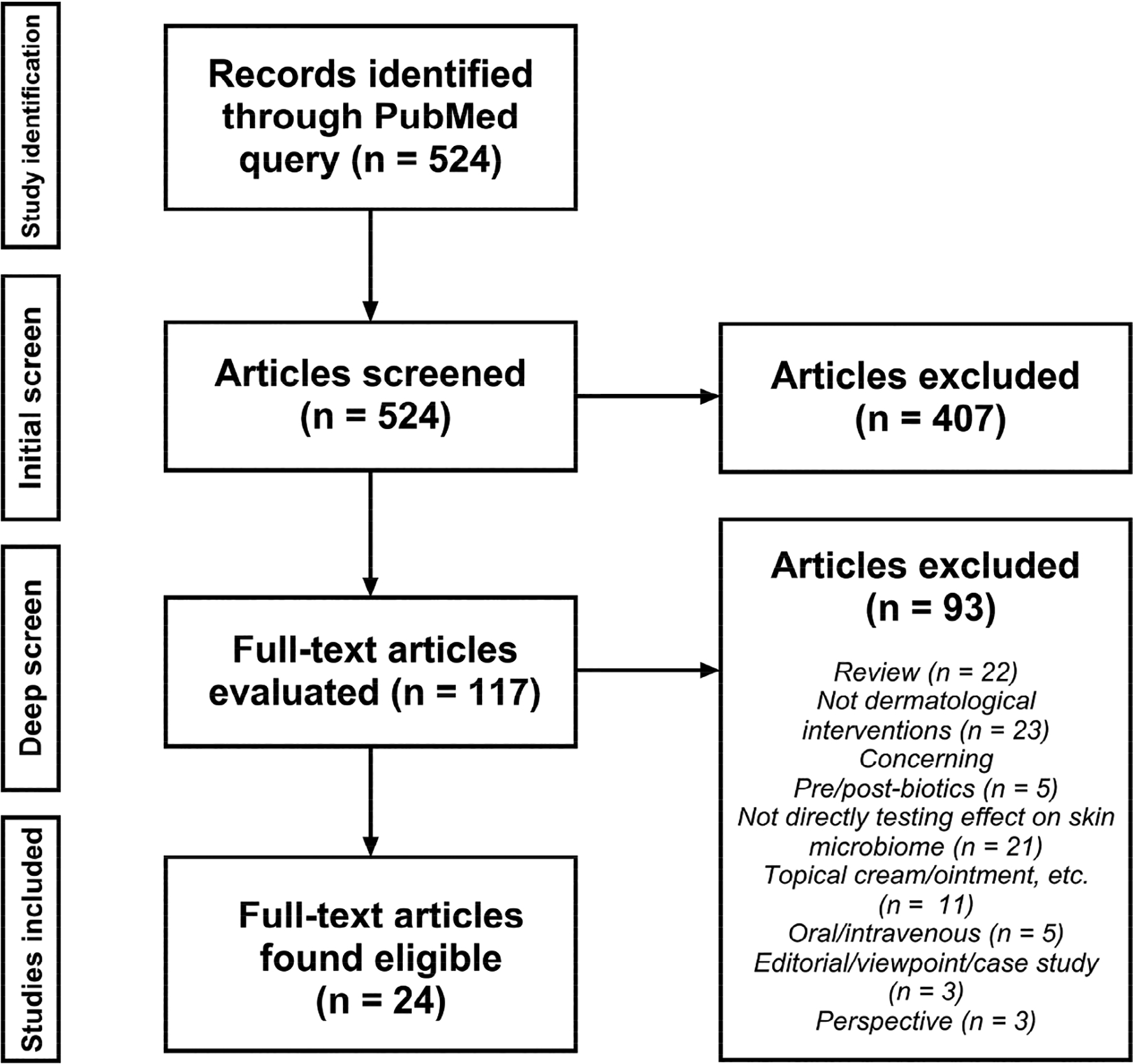
Preferred Reporting Items for Systematic Reviews and Meta-Analyses for Scoping Reviews (PRISMA-ScR) flow chart of the study selection algorithm.

The initial query identified 524 potential articles for inclusion. Title, abstract, and keyword screening excluded 407 articles that did not meet the inclusion criteria. A deep screen, full paper review led to the exclusion of 93 papers for reasons outlined in Figure 1. The items of interest were then extracted from the remaining 24 included articles.

The included articles were conducted across three distinct species, namely humans, dogs, and mice. In total, 87.5% of the articles were conducted with human subjects while 8.3% were conducted with dogs, and the same percentage with mice. Note, one article included both human and mouse subjects. The average sample size was 21.2, ranging from 5 to 125 (Tables 1 and 2). No articles studied the effects of the majority of intervention categories outlined above, specifically chemical cauterization, dermabrasion, skin grafts, cryotherapy, acne surgery, excisional biopsy, injection, electrosurgery, surgical curettement, Mohs, electrolysis, and radiation.

### 3.1. Phototherapy Significantly Alters the Skin Microbiome

We observed that over 70% of studies investigating the effects of dermatological procedures on the skin microbiome have been related to phototherapy (Table 1). This therapeutic treatment harnesses various light wavelengths to treat conditions such as psoriasis, eczema, vitiligo, and atopic dermatitis by modulating cellular processes and reducing inflammation. It includes several broad treatment types used throughout the reviewed studies—namely, UV phototherapy, light therapy, laser therapy, and photodynamic therapy [39]. It is generally associated with an increase in microbiome diversity [40–47]. However, some studies found no significant change in this regard [48,49].

UV phototherapy has been specifically useful for immune suppression and inducing DNA damage in targeted cells. It is a common treatment for a variety of conditions like psoriasis, vitiligo, and atopic dermatitis [50]. It is generally effective in reducing pathogenic bacteria, especially *Staphylococcus aureus* [41,43,51,52], while increasing the abundance of commensal *Staphylococcus* species such as *Staphylococcus capitis* and *Staphylococcus warneri* [43], and phyla such as Cyanobacteria [40,53,54]. Interestingly, Lossius et al. found no initial increase in microbiota after three narrowband ultraviolet-B (NB-UVB) treatments, but the effects did appear after 6–8 weeks of full body treatment [42]. In all cases, changes to the microbiota were present after UV phototherapy even if they were non-significant or restricted to a few organisms.

Laser therapy has been established as an appropriate way to promote wound healing and skin rejuvenation [55]. More recently it has been shown to have antibacterial effects, including suppressing growth and potentially disrupting membrane integrity and potential [56,57]. Of the three studies using laser therapy, one showed limited effects on microbial diversity but noted a decrease in *Staphylococcus pseudintermedius* [49]. Another study reported a decrease in alpha diversity [58]. The third study specifically focused on *Pseudomonas aeruginosa*, showing inhibited biofilm formation but only testing for this particular bacterium [59].

Photodynamic therapy (PDT) involves the application of a photosensitizing agent called 5-aminolevulinic acid (ALA) to the skin, which is absorbed by cells and converted into protoporphyrin IX. The treated area is exposed to specific wavelengths of light. Protoporphyrin IX absorbs the light and generates reactive oxygen species (ROS). These ROS selectively destroy the targeted cells [60]. However, this bimodal procedure of ALA application and targeted light lends itself to increased considerations when assessing the cause of microbiome impacts. Two of the three studies showed a general increase in diversity after treatment, while Guo et al. found a slight decrease in alpha diversity following treatment that decreased with subsequent treatments [44,45,61]. ALA-PDT treatment also led to the decrease of some bacteria implicated in skin diseases, such as *C. acnes*.

### 3.2. Various Dermatological Procedures Affect the Skin Microbiome

Studies on dermatology interventions other than phototherapy and their effects on the skin microbiome have been comparatively limited in scope (Table 2).

Chemical/Physical Interventions:

Chemical peels in dermatology are used to improve the skin’s appearance by treating conditions such as acne, hyperpigmentation, and signs of aging with the use of a chemical solution that exfoliates the skin and promotes cell turnover [62]. Common agents used are alpha-hydroxy acid (AHA), beta-hydroxy acid (BHA), trichloroacetic acid (TCA), and phenol. The chemical peels employed by Shao et al. (30% TCA) and Bhardwaj et al. (proprietary multi-acid synergistic peel) both showed a consistent decrease in certain bacterial populations such as *Staphylococcus* and *Propionibacterium*, although overall microbiome diversity changes varied [63,64]. Collagen masks have been purported to hydrate the skin, improve elasticity, and reduce the appearance of fine lines and wrinkles by providing a direct source of collagen to support skin structure and firmness. These masks, when used over extended periods, can increase microbial diversity indices but show negligible differences compared to untreated skin, suggesting a limited long-term impact on microbial balance [65]. The single study into high-frequency therapy showed a reduction in bacterial species and dermatophytes (Table 2), indicating its potential for targeted microbial modulation [66].

A single study observed that physical treatments, such as cosmetic piercings, modify the microbiome by decreasing *C. acnes* while increasing *Staphylococcus epidermidis*, specifically reflecting localized microbiome shifts [67]. Micropigmentation is used for cosmetic enhancement, as well as for medical applications including the camouflage of scars and vitiligo, through the precise deposition of pigment into the dermal layer of the skin. A study observing the effects of micropigmentation specifically on the cornea found no significant alteration to the ocular microbiome, suggesting minimal impact on microbial communities in this specific context [68]. Debridement, while not immediately altering the wound microbiome, reveals an association between certain bacterial genera and non-healing wounds, emphasizing the procedure’s potential influence on chronic wound outcomes [69]. Collectively, these studies illustrate the nuanced effects of various dermatological interventions on skin microbiome composition; shifts in microbiota appear to be procedure-specific. However, these findings are based on limited research, and further studies are necessary to deepen our understanding of these effects.

## 4. Discussion and Conclusions

This review highlights the scope of research regarding the impacts of dermatological procedures on the skin microbiome. The findings underscore the variable effects of different interventions, with phototherapy emerging as the most extensively studied. Phototherapy generally shows an increase in microbial diversity post-treatment. While most interventions showed some effect, phototherapy had the most pronounced effect on the skin microbiome. This is not unexpected, as light treatments have been found to kill bacteria by inducing DNA damage [71,72], producing reactive oxidative species [73–75], and damaging cellular membranes [76,77]. However, the results vary, with some studies reporting no significant changes. These discrepancies suggest that the effects of phototherapy on the skin microbiome may be influenced by factors such as the specific wavelengths of light used, the condition being treated, and individual patient differences.

The etiology of skin conditions such as psoriasis and atopic dermatitis has long been known to be related to local inflammatory processes in skin lesions of affected patients. Phototherapy is a modality employed by dermatologists to treat lesions by modulating local inflammation. This has been supported by numerous studies demonstrating the efficacy of phototherapy in reducing symptoms and improving skin appearance [78–80]. However, this presents an apparent dilemma. While phototherapy may provide an immediate benefit to these patients, if phototherapy alters the skin microbiome in a detrimental way, this may potentially mitigate short-term benefits.

For instance, Liu et al. (2021) found that patients undergoing light therapy for acne showed increases in the abundance of *Staphylococcus* species in treated skin zones [48]. This implicates the potential of post-treatment dysbiosis, which has ironically been linked to atopic dermatitis. Conversely, some studies, such as that by Hooper et al. (2022), showed that UV phototherapy treatment in cutaneous lymphoma patients led to decreases in *Staphylococcus* species, while Kwong et al. found that treatment resulted in increased microbial diversity [41,43]. This indicates that light therapy could either negatively alter the native microbiome or potentially restore it, depending on the context and conditions treated. Further research is needed to elucidate the precise mechanisms by which phototherapy affects the skin microbiome and to determine whether phototherapy could indeed cause dysbiosis, and if so, at what level.

Additionally, different wavelengths of light in phototherapy may have varied effects on the microbiome, necessitating studies to explore these variables. It is also generally understood that specific wavelengths affect bacteria in different ways, with UV and blue wavelengths generally considered to be more destructive than red or infrared light [73,81–84]. Interestingly, the studies we reviewed suggest that overall, UV light therapy results in an increase in microbial diversity and a decrease in pathogenic bacteria such as *S. aureus*, whereas laser therapy, regardless of wavelength, leads to decreased bacterial densities in all species tested [40–44,49,51–54,58,59,61]. The reasons for this are unclear, but may be due to the optical amplification innate in laser therapy. Lubart et al. found that low-level visible light is beneficial to bacterial growth while high-intensity light kills bacteria, providing a possible explanation [73].

Our review indicates that studies on dermatological interventions other than phototherapy on the skin microbiome have been comparatively limited in scope and their effect on the skin microbiome is less clear. The single study concerning piercing found a significant effect on the relative abundances of *S. epidermidis* and *C. acnes*, and the authors attribute this to the shift to a moister local environment post-piercing [67]. However, other physical treatments failed to see any significant change. Debridement was particularly surprising, as it is a method to remove unhealthy tissue, often containing pathogenic bacteria, from the skin [85]. This may be due to our inclusion protocol yielding only a single study into this treatment’s effects. Likewise, for chemical interventions, only the salicylic acid (SA) peel had a significant effect on the skin microbiome, by decreasing over-abundant *Staphylococcus* and *Propionibacterium* species in dysbiotic patients [63]. SA has previously been shown to exhibit inhibitory and bactericidal effects on S. aureus in vitro [86]. This may point to the microbiome-modulating effect in these chemical peels being due to the active ingredient (SA), and not the peel vehicle or the physical peeling action during treatment. This highlights the need for future studies investigating how physical and chemical interventions could potentially lead to dysbiosis and their long-term impacts on patients’ skin. For instance, the different chemical compositions used in peels, such as AHA, BHA, TCA, and phenol, may affect the microbiome differently. Comprehensive follow-up studies are essential for understanding the prolonged effects of these interventions.

Microbial interactions also play a large role in the dynamics between the skin microbiome and dermatological interventions. *C. acnes* has been previously shown to have an inverse relationship to *S. epidermidis* [87,88]. The studies by Liu et al. and Xu et al. observed decreases in *C. acnes* and increases in *S. epidermidis* following light therapy and piercing, respectively [48,67]. This may be an example of an antagonist relationship where the decrease in *C. acnes* was caused by the initial dermatological intervention, and the increase in *S. epidermidis* was concomitant and secondary to the lowered *C. acnes* density. Another broader relationship was found in two UV phototherapy studies which showed an inverse relationship between microbial diversity and *S. aureus* [41,43]. This may suggest that UV phototherapy reduces *S. aureus* levels, allowing for higher densities of *S. aureus* competitors. Overall, competition between species influences the makeup of the skin microbiome post-treatment.

We specifically included studies which focused on dermatological interventions that were procedural in nature using the AMA’s current CPT^®^ codes for dermatology as a start point. This provided a standardized basis for identifying which procedures to include. Interventions that consist solely of topical ointments or creams were discarded as they are not defined in the procedural codes. However, studies have found that antibiotic/antiseptic topicals in particular have caused shifts in the skin microbiome [89–91]. Likewise, topical steroids such as tacrolimus also play a role in modulating the skin microbiome in atopic dermatitis patients, with a study finding that tacrolimus treatments result in increases in skin microbiome diversity similar to that found in healthy individuals [92]. However, this study was limited due to its low sample size. Additional studies should be performed to elucidate the full scope of topical creams and ointments’ effects on the skin microbiome.

From our initial 21 CPT^®^ code-derived categories, only debridement, tattooing, micropigmentation, electrosurgery, phototherapy, laser surgery, and chemical peel were represented, with collagen masks found incidentally. This gap indicates a significant need for research across a broader range of dermatological procedures. Specifically, procedures such as cryotherapy, which drastically alters the temperature of targeted skin surfaces, could have large impacts on local microbiota. Other areas of potential research include, but are not limited to, dermabrasion, skin grafts, chemical cauterization, and radiation.

The potential of using dermatological interventions to purposefully alter and restore a healthy microbiome is an area ripe for exploration. If certain procedures can be shown to promote a balanced and diverse microbiome, they could be used to not only treat skin pathologies, but also to maintain or improve overall skin health.

Dermatological interventions are necessary measures for treating various skin conditions; however, there exists a critical need to evaluate their long- and short-term impacts on the skin microbiome, particularly regarding the potential for inducing dysbiosis. There is a large gap in knowledge surrounding the effects that many dermatological procedures have on the skin microbiome. Our review highlights the complexity of the known relationships, as evidenced by the varied responses observed across different interventions. For instance, while some studies suggest that certain treatments may lead to shifts in microbial diversity or specific taxa abundance, others indicate a potential for microbial restoration. It also highlights the need for more research into neglected dermatological procedures such as cryotherapy, skin grafts, and dermabrasion. Comprehensive follow-up investigations into whether dermatological procedures disrupt the native microbiome and precipitate dysbiosis could improve clinical practice and optimize patient outcomes in dermatological care.

## 5. Limitations

This review was conducted according to the PRISMA-ScR methodology; however, a single database, PubMed, was utilized. Additionally, the data extracted from the studies, specifically the outcomes, were qualitative and descriptive in nature, and potential errors in comprehensive extraction are therefore possible. However, two reviewers were used to mitigate error. The search terms used to capture dermatologic procedures may not be comprehensive, however, they were adapted from the AMA’s dermatology CPT^®^ codes, which is the gold standard for defining medical procedures.

## Figures and Tables

**Table 1.** Eligible studies retrieved describing the effects of light therapy on the skin microbiome, with summaries.

Author	Year	Dermatologic Procedure	Study Size (n)	Study Length	Condition	Species	Outcomes
Burns et al. [53]	2019	UV Phototherapy	6	Short-Term (24 h)	None	Human	There was an increase in *Cyanobacteria*, along with a decrease in Lactobacillaceae and Pseudomonadaceae.
Kwong et al. [41]	2019	UV Phototherapy	18 (13 were subjected to UV)	Long-Term (9 weeks)	Atopic Dermatitis	Human	Microbial diversity in lesional skin increased after treatment. The *Staphylococcus aureus* proportion decreased with treatment.
Lossius et al. [42]	2021	UV Phototherapy	16	Long-Term (6–8 weeks)	Atopic Dermatitis	Human	Lesional AD skin microbiota showed higher diversity after 6–8 weeks of treatment, while NLS and nose/throat microbiota remained unchanged. No significant changes in microbiota were observed after only three NB-UVB treatments.
Hooper et al. [43]	2022	UV Phototherapy	40 (25 exposed, 15 control)	Long-Term (~6.2 months)	Cutaneous Lymphoma	Human	Microbial diversity increased in NB-UVB responders. The relative abundance of *S. aureus* and *Staphylococcus lugdunensis* was reduced post-treatment. Higher levels of *Staphylococcus capitis* and *Staphylococcus warneri* were recorded in responder lesional skin before NB-UVB. Decreased *S. aureus* and increased *S. capitis, Staphylococcus hominis, Staphylococcus pettenkoferi*, and *S. warneri* levels were found in responder skin post-treatment. *Staphylococcus* species abundance is more similar between non-responders and non-NB-UVB patients than between responders and non-NB-UVB patients.
Assarsson et al. [51]	2018	UV Phototherapy	26	Long-Term (10.5 weeks)	Chronic Plaque Psoriasis	Human	Increased relative abundance of *Clostridium* and decreased relative abundance of *Pseudomonas* occurred in both lesional and non-lesional skin, along with increased *Megasphaera* in non-lesional skin.
Dotterud et al. [52]	2008	UV Phototherapy	40 (20 dermatitis, 20 control)	Long-Term (6 weeks)	Atopic Dermatitis	Human	*S. aureus* counts in lesional skin showed a non-significant decrease after 4 weeks of treatment, with a slight increase observed after a 2-week follow-up. Similar trends were observed in non-lesional skin and the forehead.
Wang et al. [61]	2012	UV Phototherapy	5 human, 5 mice	Short-Term (1 day)	None	Human and Mouse	Reduced porphyrin production occurred in human facial bacteria and in *Cutibacterium acnes*-inoculated mouse ears.
Yuan et al. [44]	2020	UV Phototherapy	60	N/A	Vitiligo	Human	The NB group showed significantly higher diversity indices compared to NF, while the NF and DB groups did not differ significantly. *Staphylococcus, Bacillus*, and *Prevotella* were enriched in DF compared to DB, while *Propionibacterium* showed the opposite trend.
Park et al. [40]	2021	UV Phototherapy	20 (10 with atopic dermatitis, 10 without)	Long-Term (2 months)	Atopic Dermatitis	Dog	Phototherapy altered the skin microbiome in dogs with AD, increasing Actinobacteria and Cyanobacteria and decreasing *Staphylococcus pseudintermedius*. Higher alpha diversity occurred after treatment.
Kurosaki et al. [54]	2020	UV Phototherapy	22 (11 lesional, 11 non-lesional)	Long-Term (2 months)	Atopic Dermatitis	Human	An increase in Cyanobacteria and a decrease in Bacteroidetes occurred in lesional skin. A significant reduction in the abundance of *S. aureus* was also found at the species level in lesional skin.
Liu et al. [48]	2021	Light Therapy	39 (20 acne, 19 healthy)	Long-Term (3 months)	Acne	Human	There was a significant increase in the relative abundance of *Staphylococcus epidermidis*, while *C. acnes* decreased.
Munoz Declara et al. [49]	2024	Laser Therapy (905 nm, 808 nm)	20	Short-Term (6 days)	Atopic Dermatitis	Dog	No significant alterations in microbiome composition or diversity were observed, but a decrease in the relative abundance of *S. pseudintermedius* was noted in the treated areas of some dogs.
Park et al. [58]	2023	Laser Therapy (755 nm)	21	Long-Term (3 months)	Rosacea	Human	There was a decrease in the relative abundance of *Cutibacterium*, *Streptococcus*, *Clostridium*, *Bacteroides*, and *Lactobacillus*. An increase in the relative abundance of *Staphylococcus*, Neisseriaceae, *Corynebacterium*, *Anaerococcus*, and *Lawsonella* also occurred. There was a decrease in alpha diversity after treatment.
Rupel et al. [59]	2019	Laser Therapy (445 nm)	15 (8 treatment, 7 control)	Short-Term (single application)	None	Mouse	Blue laser light decreased *Pseudomonas aeruginosa* both in vitro and in vivo. This inhibited biofilm formation.
Guo et al. [45]	2022	ALA Photodynamic Therapy	26 (18 with acne, 8 without)	Long-Term (3 weeks)	Acne	Human	Reduced alpha diversity occurred after treatment. There was no statistically significant difference observed among different groups for *C. acnes* at the genus level. There was an increase in the abundance of *Pseudomonas*, *Gordonia*, *Leptotrichia*, and *Mycobacterium*, restoring them to healthy levels.
Yang et al. [46]	2021	ALA Photodynamic Therapy	5	Long-Term (2 months)	Acne	Human	PDT inhibited *C. acnes* in the follicular microbiome. *Bacillus* and *Lactococcus* increased post-PDT. ALA-PDT increased microbiome diversity and made the follicular microbiome more like the epidermal microbiome taxonomically and functionally.
Tao et al. [47]	2021	ALA Photodynamic Therapy	11	Long-Term (6 weeks)	Acne	Human	There was a notable decrease in the relative abundance of C. acnes, whereas *Pseudomonas fluorescens* significantly increased. No effect on *S. epidermidis* was found. Additionally, ALA-PDT was correlated with heightened microbiota diversity and reductions in the relative abundance of functional genes related to energy metabolism and DNA replication.

NB-UVB = narrowband ultraviolet-B; AD = Atopic Dermatitis; NF = normal skin prior to radiation; NB = normal skin after radiation; DF = lesional skin prior to radiation; DB = lesional skin after radiation; ALA = 5-Aminolevulinic acid; PDT = photodynamic therapy; NLS = non-lesional skin.

**Table 2.** Eligible studies retrieved describing the effects of chemical and physical dermatologic procedures on the skin microbiome, with summaries.

Author	Year	Dermatologic Procedure	Study Size (n)	Study Length	Condition	Species	Outcomes
Shao et al. [63]	2023	Chemical Peel	28	Long-Term (2 months)	Acne	Human	*Staphylococcus* and *Propionibacterium* proportions tended to decrease.
Bhardwaj et al. [64]	2024	Chemical Peel	9	Short-Term (single application, 20 min)	Hyperpigmentation	Human	Non-significant raise in Shannon’s diversity index, a mathematical measure of species diversity within a community [70]. Beta diversity remained constant. No change in the abundance of *Staphylococcus epidermidis*. Reduction *in Cutibacterium acnes*. Decreased Porphyrin.
Janssens-Bocker et al. [65]	2024	Mask	28	Long-Term (4 weeks)	None	Human	Shannon’s diversity index significantly increased from baseline, but showed no difference compared to untreated areas. The genus *Staphylococcus*, as well as *S. epidermidis*, specifically decreased significantly over time, but not compared to untreated areas. No significant changes were observed for *Corynebacterium, Pseudomonas*, or *C. acnes* between time points or compared to untreated areas.
Frommherz et al. [66]	2022	Electrotherapy (HF therapy)	N/A	N/A	Acne	N/A	Bacterial species decreased, including *Aerococcus viridans*, *Bacillus cereus, Aerococcus urinaeequi, Staphylococcus lugdunensis, Staphylococcus haemolyticus*, *Micrococcus yunnanensis*, *Micrococcus luteus*, and *Mycobacterium* species. Dermatophytes decreased in colony count post-HF treatment, including *Trichophyton benhamiae*, *Trichophyton rubrum*, *Trichophyton mentagrophytes*, *Trichophyton violaceum*, and *Microsporum canis*.
Xu et al. [67]	2023	Piercing	28	Long-Term (2 weeks)	None	Human	Decrease in the relative frequency of *C. acnes* and a significant increase in the relative frequency of *S. epidermidis* in the piercing microbiome.
Yilmaz et al. [68]	2023	Micropigmentation	125 (35 corneal tattoos, 40 corneal leukoma, 50 healthy)	Unknown	Corneal Leukoma	Human	No significant difference between native and tattooed eyes.
Verbanic et al. [69]	2020	Debridement	20	Short-Term (immediately after debridement)	Chronic Wound	Human	Sharp debridement did not directly alter the wound microbiome compared to the original wound surface. However, aerobes and facultative anaerobes, particularly the genus *Enterobacter*, were significantly associated with wounds that did not heal within 6 months.

HF = High-frequency.
